# Student nurse education and preparation for palliative care: A scoping review

**DOI:** 10.1371/journal.pone.0286678

**Published:** 2023-07-03

**Authors:** Abiola Durojaiye, Ruth Ryan, Owen Doody

**Affiliations:** 1 Department of Nursing and Midwifery, University of Limerick, Limerick, Ireland; 2 Department of Nursing and Midwifery, Health Research Institute, University of Limerick, Limerick, Ireland; Imam Abdulrahman Bin Faisal University, SAUDI ARABIA

## Abstract

**Background:**

The World Health Organisation and palliative care stakeholders recommend that healthcare workers are educated in palliative care. Provision of high-quality palliative care is fundamental to nursing practice. However, caring for palliative care patients and meeting family needs is challenging without appropriate knowledge and experience. Palliative care education and clinical skill development for undergraduate student nurses is a priority to ensure graduate nurses are equipped with the knowledge and skill to deliver safe and competent care.

**Methods:**

A scoping review guided by Arksey and O’Malley’s framework was used to identify undergraduate student nurses’ palliative care education and preparation. A comprehensive literature search of five electronic databases and grey literature were conducted from January 2002 to December 2021. The aim was to review the empirical evidence and ascertain how undergraduate student nurses’ palliative care education is organised, facilitated, delivered and evaluated. Screening was performed independently by two reviewers against eligibility criteria with meetings to discuss included papers and form a consensus. Data was extracted and related to palliative care undergraduate student nurses’ education, educational model, methodology, key findings, and recommendations. Analysed and summarised data was mapped onto the four key review questions (educational models utilised, methods used to assess effectiveness, facilitators/barriers and gaps in the literature).

**Results:**

34 papers met the criteria for this review. The review highlights that undergraduate nursing palliative care education is more evident in high income countries. Limited and diverse published research existing in low- and middle-income countries. Educational models utilised were theoretical and experiential learning and educational process, early integration and multiple learning methods which were highlighted as facilitating factors. However, crowded curricula, lack of palliative care clinical placement expertise, difficulty providing clinical placement, timing and delivery of palliative care and difficulty responding to simulated environments (manikins) were perceived barriers. Nevertheless, palliative care education can increase knowledge, positive attitude, self-confidence and adequate preparation of undergraduate student nurses.

**Conclusion:**

This review highlights that there is limited research regarding the timing and delivery of palliative care principles and practice in undergraduate student nurse education. Early integration of palliative care education impacts upon students perceived preparedness for practice and positively influences their attitudes to palliative care provision.

## Background

Internationally, nurse education has and continues to experience radical change in response to societal demands and professional requirements and standards. The move from medical task-oriented care to individual person and family-centred care at all stages of the lifespan is reflected in the education models that underpin the art and science of the nursing profession [1]. Focusing on palliative care is an essential part of nursing education and it is important for undergraduate student nurse to gain knowledge on palliative care to improve the overall quality of healthcare [2]. The need for palliative care education has been well documented by the World Health Organisation [3] and supported by the International Council of Nurses [4]. This need highlights that we require undergraduate student nurses to be appropriately educated to have the necessary knowledge, skills and attitudes to be able to provide palliative care across all healthcare settings upon graduation. Palliative care encompasses:

“*the active holistic care of individuals across all ages with serious health-related suffering due to severe illness, and especially of those near the end-of-life. It aims to improve the quality of life of patients, their families and their caregivers” [5]*

The elimination of serious health-related suffering includes the various health conditions or severe illness that are most likely to generate a need for palliative care. Health-related suffering is considered serious when it compromises physical, social, spiritual, and/or emotional functioning, and when it cannot be relieved with professional intervention. The goal of palliative care is to prevent and relieve suffering, and to support the best possible quality of life for patients and their families, regardless of the stage of the disease or the need for other therapies [5]. The alleviation of suffering is an essential function of nursing [6] and caring for a patient that requires palliative care and meeting the family’s needs is challenging without appropriate knowledge [7]. Educational and clinical training requirements for undergraduate student nurses in palliative care is a phenomenon that must be considered globally. A limited but growing number of schools of nursing programmes include palliative care content in their undergraduate nursing curricula [8, 9]. In addition, consensus conferences and position statements have been developed to outline and define the attitudes, knowledge and skills involved in palliative care [8, 9].

While in 1967 Cicely Saunders [10] made a seminal contribution to palliative care philosophy and practice there has been a slow gradual growth and development of palliative care education. Before the 1990s, the palliative care educational needs of nurses were often overlooked in traditional nursing education settings [6]. Post 2002, there has been a great focus and emphasis for the need of palliative care education within undergraduate nursing programs. This focus and development can be linked to the priority and definition given to palliative care by the WHO [11]. Following the WHO definition in 2002 many palliative care programmes have been integrated in nursing curricula in the Western world. Palliative care educational programs that have been described typically consist of multisession training programs that incorporate attitudinal discussions and provide core knowledge and skills practice. According to the WHO policy on palliative care education, it is expected that palliative care education would be embedded in all nursing programs, but this remains unrealised in some schools of nursing [9]. Teaching palliative care within undergraduate student nurse education is seen to improve holistic, compassionate, individualised person and family-centred care [1]. Therefore, there is a need to investigate how palliative care education is embedded in undergraduate nursing curriculum, what is being taught, how it is delivered, and how undergraduate student nurses are prepared for clinical practice. Furthermore, as compassionate, competent and holistic care are at the core of nursing, knowledge of palliative care concepts is a fundamental expectation of graduating nursing students [12]. The shared theory of palliative care [13] suggests that to be competent in providing palliative care one requires sufficient knowledge and as competence influences performed interventions this translates to patient outcomes. This relationships between knowledge, competence, and patient outcomes provide a clear rationale to address the aspect of undergraduate nurses’ palliative care education. While reviews have been conducted within undergraduate nurse education, they have focused on educational interventions [14], effects of simulation [15], modes of delivery/teaching strategies [16] and death education [17]. In addition, this work was pre-1994 [17] and between 1984–2012 [16], 2000–2013 [14] and 2011–2016 [15]. Thus, there is a need to map the literature to identify undergraduate student nurses’ palliative care education and preparation for their practice as a registered nurse. This paper addressed this need through reviewing the literature from 2002 to 2021 representing the 20 years since the WHO 2002 definition of palliative care.

## Methods

Due to the broad nature of palliative care and nursing education, a scoping review methodology was employed. This allowed for the presentation of a broad synthesis and mapping of the available evidence which is not limited by study quality or design [18]. This was an interactive process where each step was returned to and advanced during the process [19]. Consequently, utilising a scoping review assisted in identifying the current body of knowledge and existing gaps in the literature [20]. Through the systematic and transparent synthesis of the evidence, a rigorous map of the findings is presented in order to highlight the extent and nature of the literature, identify gaps and make recommendations [21, 22]. The Arksey and O’Malley [18] framework was adopted for this review and the authors incorporated recent scoping review methods updates [21–23].

The framework utilises a five-step process:

- identifying the research question,
- identifying relevant studies,
- study selection,
- plotting the data, and
- arranging, summarising and communicating the outcomes

### Aim

The aim of this scoping review is to scope and map the literature to identify undergraduate student nurses’ palliative care education and preparation for practice.

### Identification of research question

Step one of Arksey and O’Malley’s [18] framework and to meet the aim of this review, the focus is on the following questions:

- What educational models are utilised within palliative care undergraduate student nurse education?
- What methods have been used to assess effectiveness of palliative care undergraduate student nurse education?
- What facilitators or barriers have been reported relating to the success/failure of the models of palliative care undergraduate student nurse education?
- What gaps in the literature exist on preparation within palliative care undergraduate student nurse education?

### Identification of relevant studies

In step two of Arksey and O’Malley’s [18] framework to capture the broad scope of palliative care literature, a broad range of keywords and MeSH terms were used within the search [18]. The search strings were developed and agreed by the review team (AD/RR/OD). A search strategy (Table 1) and inclusion criteria guided the review, and the search was conducted across five databases MEDLINE, Cumulative Index to Nursing and Allied Health Literature (CINAHL), Academic Search Complete, PsycINFO and Cochrane Library. An additional search was conducted for grey literature (International Clinical Trials Registry Platform Search Portal) across nursing and palliative care websites. The search was conducted from the 01-01-2002 to the 10-12-2021. The search words were used in ‘title’ and ‘abstract’ searches utilising Boolean operators ‘OR’ and search strings were finally combined using Boolean operators ‘AND’. All citations were exported to Endnote Library 2021 (Clarivate Analytics, Pennsylvania, USA) for duplicates to be identified and removed (AD/OD) and exported to Rayyan (Qatar Computing Research Institute) for screening and voting to occur (AD/OD).

**Table 1:** Database search.

	Search terms
S1	Nurs* OR education OR under-graduate OR undergraduate OR under graduate OR pre-registration OR bachelor OR diploma OR preregistration OR pre-registration OR pre license OR baccalaureate OR (MM “Nursing+”) OR (MM “Students, Nursing”)
S2	palliative care OR terminal OR life limiting OT life-limiting OR life threatening OR dying OR (MM “Palliative Care”)
S3	program* OR programme* OR education OR prepar* OR train* OR teach* OR (MM “Education+”) OR (MM “Education, Nursing+”)
S4	S1 + S2+ S3

### Study selection

For the third step of Arksey and O’Malley’s [18] framework papers were screened in Rayyan independently by two reviewers to identify papers that meet the selection criteria (Table 2). Then, the full texts of the remaining studies were retrieved and screened against the selection criteria. Papers that met the selection criteria were included in the review.

**Table 2:** Inclusion and exclusion criteria.

Inclusion Criteria	Exclusion Criteria
Published between 01-01-2002 and 09-12-2021. This is to accommodate palliative care definition by WHO, 2002.	Papers published outside the search timeframe.
English language	Languages other than English.
Undergraduate palliative care education delivered by higher education institutes.	Continuing professional education modules or postgraduate programme/s or in-service training or hospital training programme/s
Papers with mixed samples but possible to extract data pertaining to undergraduate nursing education.	Papers where it is not possible to extract data pertaining to undergraduate nursing education.
All study types and grey literature.	

### Mapping/plotting of data

The fourth step of Arksey and O’Malley’s [18] framework involved mapped the existing literature in terms of nature, characteristics and source of evidence [24]. In accordance with Arksey and O’Malley’s [18] process, this stage involved extracting summaries from each paper in a data extraction table (Table 3). The data extracted from each paper pertained to the author, year, title, country, aim/focus of the paper, educational model, methodology, key findings and recommendations for undergraduate nursing palliative care education and practice. The extracted data supported the mapping of data onto the review questions and to meet the aim of the review.

**Table 3:** Data extraction table.

Author(s) Year Title Country	Aim/Focus	Educational model	Methodology	Findings	Recommendations
[25] Alt-Gehrman, (2017) Education provided for nursing students about end-of-life care. United States of America.	To determine what is provided to undergraduate nursing students regarding EOL.	Conceptual framework not evident. Year of students’ program: Undergraduate nurses. Facilitation not evident. Delivery by lecture, simulation and experiential methods.	Literature review. 2000 to 2017 14 papers Themes generated.	Multiple methods of education delivery have shown to increase nursing students’ knowledge about EOL care, and a positive attitude can affect the care delivered at EOL.	Recommendation for education and practice not evident.
[14] Bassah et al., (2014). A Modified systematic review of research evidence about education for pre-registration nurses in palliative care. United Kingdom.	To examine educational approaches applied to pre-registration palliative care nursing education and their effectiveness.	Conceptual framework, year of students’ program and facilitation not evident. Both didactic and experiential educational strategies either as a discrete course or embedded in other specialty nursing courses.	Modified Systematic review. January 2000 to December 2013. 17 papers. Method of analysis not evident.	Lack of competence and confidence in providing palliative care.	There is need for pre-registration student nurses to be educated in palliative care, prior to entering the professional practice. Suggestions are made for the development of preregistration PC education in resource poor countries.
[26] Birkholz et al., (2004) Students’ self-identified learning needs: a case study of baccalaureate students designing their own death and dying course curriculum. United States of America.	The paper describes a class of honours nursing students who identified their own learning needs and developed a course on death and dying.	Conceptual framework not evident. Year of students’ program: Three-year course baccalaureate students. Facilitation by guests’ lecturers, religious leader, ethicist, social worker, counsellor, psychotherapist, morticians, nurses, physicians, lawyer, caregivers, and classmate from a different culture. Delivered through classroom didactic and experiential approaches.	Ethic approval not evident. Qualitative study Non-probability sampling. 17 female junior and senior baccalaureate students. Data collected through class summary.	Students gained more knowledge in helping patients, families, and themselves in end-of-life care related issues.	Nursing students recognised the need for adequate EOL education that is both didactic and experiential.
[27] Carmack and Kemery, (2018). Teaching methodologies for end-of-life in undergraduate nursing students. United States of America.	To explore current strategies, compare findings to that of previously published results, and determine whether progress has been made in the interim.	Conceptual framework, year of students’ program and facilitation not evident. Delivery strategy face-to-face instruction, clinical or practicum experience, online teaching, simulation and interprofessional education.	Literature review. January 2011 to May 2016. 22 papers.	Preparedness and implication on practice not evident.	More attention should be paid to clinical and online education outcomes. Future research into the efficacy of EOL teaching strategies should include an intervention, rather than rely on survey. Future studies should focus on a single intervention or include enough for comparison or intervention groups. Use of standardized tools in future research.
[28] D’Antonio, (2017) End-of-life nursing education. Past and present. United States of America.	Focus on the history, development, and teaching methods of EOL care and offers recommendations for future education.	Conceptual framework and year of students’ program not evident. Facilitation is by nurse educators and expert guests’ lecturers. Delivery methods include lecture, seminar/ small group format, role-play, videos case studies, and visits to local hospices and/or terminal patient visits and online format.	Papers discusses the historical development of EOL teaching model.	Preparedness not evident. However, implication on practice: Debriefing and analysis of the dynamics occurring during the scenario are an important component for students when simulation is used.	Education in EOL care should include nurse externships, tailor-made orientations, and thoughtful selection of expert mentors and preceptors.
[29] Davis et al., (2021). Integrating the ELNEC undergraduate curriculum into nursing education: lesson learned. United States of America.	To describe the benefits to schools of nursing of adopting the ELNEC UG curriculum, discuss the barriers faculty have faced regarding adoption, and offer schools strategies to help them overcome such barriers.	Conceptual framework, year of students’ program and facilitation not evident. Paper summarized some educators experience of using online platform interactive technology and designed to support faculty with and without palliative care expertise.	Ethical approval not evident. Descriptive design. Documentary review. Findings from respective schools was by formative evaluation processes.	Integration of undergraduate ELNEC throughout nursing education had enhanced undergraduate student nurses’ comfort in providing end-of-life care as new nurses. Early integration impacted student perceived preparedness and attitudes positively.	Comprehensive palliative care education must include not only didactic components, but practice-based experiences as well. Best practice to integrate PC course is to engage key faculty stakeholders, offer palliative curriculum as an independent course, utilise clinical preceptors and clinical experiences to include debriefing of the content in the clinical conference time.
[30] Davis et al., (2020) Development of a new undergraduate palliative care knowledge measure. United States of America.	To describe development and psychometric evaluation of a new palliative care measure.	The Shared Theory of Palliative Care guided the theoretical foundation. Year of students’ program: junior and senior pre-licensure baccalaureate students. Facilitation by palliative care educators. Delivery not evident.	Ethical approval not evident. Exploratory descriptive design. The Undergraduate Nursing Palliative Care Knowledge Survey (UNPCKS) used to assess primary palliative care knowledge of students. Data analysed by exploratory factor analysis. Intervention: shared Theory.	Preparedness and implication on practice not evident.	To address desirability and acceptability of adopting UNPCKS, future research should examine the burden of time imposed on both faculties to administer as well as for students who already experience much testing.
[31] Downing, (2006) Palliative care education in Uganda. Uganda.	Aim not evident. Focus is on palliative care practice and institutions providing palliative care education for healthcare teams in Uganda.	Conceptual framework N/A. Year of students’ program, facilitation and delivery not evident.	Discussion paper.	Preparedness and impact on practice not evident but discussion stated lack of knowledge about palliative care amongst nurses.	Education and care go hand-in-hand with training in palliative care is one way to break down some of the barriers of lack of knowledge.
[32] Eltaybani, (2021) Palliative and end-of-life care education in prelicensure nursing curricula: a national survey in an Arab country. Egypt.	To identify what PEOL care education is delivered to undergraduate nurses in Egypt and the teaching strategies used to deliver this education. To assess the feasibility of using online surveys in nursing research in Egypt.	Conceptual framework and year of students’ program not evident. Facilitation by nurse educators. Delivered by lecture pedagogy teaching strategy, clinical field practice, and group discussion respectively.	Ethical approval by administrative authorities and Research Ethics Committee of the Graduate School of Medicine, University of Tokyo, Japan. Cross-sectional survey. Random sampling. 88 nurse educators in the faculties of nursing. Data collected using online survey.	Preparedness not evident. Students can be exposed to meaningful learning opportunities in medical-surgical and intensive care units in local hospitals and health centres that provide care to patients with serious, life-threatening illnesses.	Evidence-based program is recommended to guide the development of PEOL care discrete and sufficient course contents for nursing students. Utilization of simulation using high-fidelity mannequins is also perceived as an effective teaching strategy. Training nursing educators) on maximizing the use of high-fidelity simulation to teach PEOL care may help to overcome the scarcity of specialized PC institutions across the country.
[33] Ferrell, (2018) An Innovative end-of-life nursing education consortium curriculum that prepares nursing students to provide primary palliative care. United States of America.	The paper describes the development and implementation of an innovative online nursing curriculum that prepares students with essential primary palliative nursing knowledge and skills.	Conceptual framework not evident. Year of students’ program: Pre-licensure students. Facilitation by palliative care nurse leaders. Delivered through case studies with critical-thinking reflection, brief videos demonstrating key palliative nursing skills, and 2 to 3 nursing licensure examination style questions woven throughout. Students are required to complete a 10-question quiz with nursing licensure type items.	Discussion paper.	Students have primary palliative care contents in their curricula and felt more prepared to care for patients and families, especially at the EOL.	Faculties are encouraged to see it as a privilege to educate future nurses in palliative care so they can provide high quality primary palliative care.
[34] Ferrell et al., (2016) CARES: AACN’s new competencies and recommendations for educating undergraduate nursing students to improve palliative care. United States of America.	An historical manuscript and CARES document that focuses on content areas that should be included in palliative nursing education.	Conceptual framework, year of students’ program and facilitation and delivery not evident.	Historical documentary review.	Student preparedness and implication on practice not evident.	PC nurse education content should be integrated into a fundamentals / introduction to nursing course in which topics such as pain, comfort, communication, and care of the imminently dying patient are covered.
[35] Glover et al., (2017) An experiential learning approach to primary palliative care nursing education: the comfort shawl project United States of America.	The paper focus explains students’ participation in numerous experiential learning activities during the Comfort Shawl.	Conceptual framework and year of students’ program not evident. Facilitation: teacher led. Delivery: experiential activities, including touring the local hospice care centre and retirement community.	Discussion paper.	Preparedness and impact on practice not evident.	Clinical experiences in palliative care are vital to supplement didactic learning.
[36] Goode et al., (2019) Person-centred end-of-life curriculum design in adult pre-registration undergraduate nurse education: a three-year longitudinal evaluation study. United Kingdom.	To explore student evaluation of end-of-life care learning within a three-year undergraduate adult nursing degree programme.	Conceptual framework Scaffolded approach. Year of students’ program: Third-year nurses. Facilitation not evident. Delivery is by pedagogical approaches to encourage problem-based learning, facilitating role play and exploration of audio-visual resources.	University ethics committee approval. A longitudinal quantitative approach containing open and closed questions. 336 Third-year students. Intervention: scaffolded approach.	Students identified growth in their practice, confidence and preparedness to deliver person-centred care in end-of-life-care. However, they requested for more use of scenarios, communication and practical application in year one.	Ensure structured and scaffolded learning across programmes of study. Learning experience impacts on both professional practice and personal life requires appropriate consideration during the planning of teaching students. Involvement of voluntary and independent sectors, primary and secondary social care provider in planning and delivery of EOL education.
[37] Heath et al., (2021) Preparing nurses for palliative and end of life care: a survey of New Zealand nursing schools. New Zealand.	To explore teaching content, organisation, delivery, assessment and clinical learning opportunities in palliative and end-of-life care in undergraduate nurse education in New Zealand.	Conceptual framework not evident. Year of students’ program not evident. Facilitation by academic nurses with contributions from other professionals. Delivery by lectures, tutorials Clinical placements (generally of 1 week or more), case-based teaching, simulation and patient presentation / interview.	Ethical approval not evident. Quantitative descriptive cross-sectional study. 13 academic leads out of 18 educational institutes participated. Quantitative–online cross-sectional survey.	Palliative care is included in undergraduate teaching.	Need to address the time demands within curriculum to meet all criteria. Lack of clinical placements and inconsistencies needs to be addressed. Specific palliative care assessment should be incorporated into programmes.
[38] Henoch et al., (2017) Undergraduate nursing students’ attitudes and preparedness towards caring for dying persons: a longitudinal study. United Kingdom.	To describe the development of nursing students’ attitudes toward caring for dying patients and their perceived preparedness to perform end-of-life care.	Conceptual framework not evident. Year of students’ program: Third-year nursing students. Facilitation not evident. Delivery by simulation.	Ethics approved by the Regional Ethics Committee, and the head of nursing program of each institution. A longitudinal study of 117 nursing students participated and completed the Frommelt Attitude Toward Care of the Dying Scale (FATCOD).	Simulation was said to adequately prepared student nurses for EOL care. Structured palliative care course influenced students’ ability to be clinically competent.	There is a need for the palliative care component to be at least five weeks in length for it to have an impact. Communication reflection, and interactive exercises are recommended to help student nurses more prepared for the care of the dying patients.
[39] Hjelmfor et al., (2016). Simulation to teach nursing students about end-of-life. United States of America.	To increase the knowledge about end-of-life care simulation in nursing education by describing and evaluating the delivery of simulation when teaching third-year nursing students about end-of-life care.	Conceptual framework not evident Year of students’ program: 3^rd^ year nursing students. Facilitation is by nurse tutors. Delivery by simulation.	Ethics not evident. Students gave verbal consent. Ethnography observation of 60 students in eight group sessions. Data collection by audio and video recorded.	Majority of the students expressed the usefulness, realistic and a good learning opportunity to handle challenging communication from patients and family members.	There is need for the faculty to bring in tutors who are nurses with a vaster experience and competence in palliative care to take part in the simulation sessions and the debriefings, to bring a greater knowledge of palliative care into nurse education.
[40] Jacono et al., (2011). Developing Palliative Care competencies for the education of entry level baccalaureate prepared Canadian nurses. Canada.	To contribute to knowledge about generating national consensus-based competencies, and to disseminate the competencies.	Conceptual framework, year of students’ program, facilitation and delivery not evident.	Ethics N/A Multiple consultation of symposium and consensus building process. Sample: directors from 8 faculties of Canadian Schools of nursing, palliative care, physicians and social worker representatives. Consultation survey via telephone and email.	Preparedness and impact on practice not evident. Barrier identified include an already crowded program. Lack of PEOLC expertise among faculties. Difficulty providing appropriate clinical placements.	Recommendation for education and practice not evident.
[41] Jianga et al., (2019) Attitude and knowledge of undergraduate nursing students about palliative care: An analysis of influencing factors. China.	To investigate undergraduate nursing students’ knowledge about and attitudes toward palliative care and analyse their influencing factors.	Conceptual framework, year of students’ program, facilitation and delivery not evident.	Ethics committees of the Universities approval. A descriptive research method that used cross-sectional survey. A stratified random sampling method used to select 1200 undergraduate nursing students from four medical universities.	Knowledge about palliative care among Chinese nursing students was insufficient.	The study suggested that additional PC training and medical universities should set up an individual palliative and EOL care curriculum for nursing students as early as possible. It was recommended that religious beliefs should be honoured and integrated.
[42] Johnson et al., (2009) Nursing the dying: a descriptive survey of Australian undergraduate nursing curricula. Australia.	To gain some insights into the relative adequacy of Australian undergraduate nursing programmes to prepare nurses to deliver care to the dying patient.	Conceptual framework N/A Year of students’ program not evident. Facilitation by registered nurses from the palliative care clinical setting and delivery is by didactic lecture format, experiential strategies and formal lecture.	The University Ethics Committee approval. A descriptive survey study. 36 Deans of nursing and midwifery schools. Survey through email.	Preparedness and impact on practice not evident.	Behavioural aspects of caring for the dying, and urgent attention of relevant theoretical content in sufficient depth combined with teaching strategies that promote critical reflection in undergraduate nursing programmes is essential to be embedded in the curricula.
[43] Karkada et al., (2011) Awareness of palliative care among diploma nursing students. India.	To identify the level of knowledge and attitude of nursing students towards palliative care who are future caretakers of patients, which helps to make recommendations in incorporating palliative care concepts in the nursing curriculum	Conceptual framework not evident. Year of students’ program: Third year diploma nursing students. Facilitation and delivery not evident.	Administrative permission obtained from the principals of selected schools. A correlative survey. Cluster sampling method. Verbal consent obtained from the study participants. 83 third-year diploma nursing students. A cross-sectional correlative structured and validated questionnaire survey and cluster technique.	Majority (79.5%) of third year nursing students surveyed had poor knowledge on palliative care but they had a favourable attitude towards palliative care. Preparedness and impact on practice not evident.	PC aspects such as patient-centred communication, ethical issues, decision making at the EOL, whole person care and interdisciplinary work are important and can have a lasting impact on future health practice. To be a competent student nurses need to be prepared to take care of the terminally ill patient at the grassroot level through home visit.
[44] Kirkpatrick et al., (2017) Palliative Care Simulation in undergraduate nursing education: an integrative review. United States of America.	To explore the effects of simulation-based learning experience on nursing students’ preparation to delivery palliative care.	Conceptual framework, year of students’ program and facilitation not evident. Delivery via simulation (high-fidelity simulators/role play/actors). Roles assigned (active, observer etc). Observer checklist used for debriefing sessions.	Integrative review. 2011 to 2016. 19 papers. Emerging themes.	The use of live actors and role play were effective in increasing student knowledge and self-efficacy. Students can find it difficult responding to a manikin and are more engaged when a person enacts the patient role. Presence of family members and multicultural and spiritual traits enhanced the realism of the simulation. Simulations increased confidence, communication skills, reassurance, understanding of the complexity and priorities of palliative care principles.	Learning more realistic when use standardized patients. Debriefing affords the opportunity for reflection, feedback and learning. Theoretical frameworks to underpin palliative care nursing education required.
[45] Knopp de Carvalho et al., (2017) Educational process in palliative care and the thought reform. Brazil.	To know the contributions of the educational process in Palliative Care during the undergraduate level for the professional action of nurses in the care of patients at the end-of-life.	Morin’s Theory of Complexity. Hologrammatic principle of complex thinking. Year of students’ program and facilitation not evident. Delivery by reflection on issues.	Ethics approval by Research Ethics Committee. A qualitative approach. Purposive sampling. Data collected from 13 participants (7 newly trained nurses and 6 nurse tutors) through a semi-structured interview.	Educational process in a PC education has contributed to preparing students for the care of terminally ill patients.	It is critical that education is oriented toward building awareness that all PC components are important and need to be involved in care. It is important to use relevant therapies to relieve their signs and symptoms.
[46] Li et al., (2019) Undergraduate nursing students’ perception of end-of-life care education placement in the nursing curriculum. China.	Aim not evident. Focus is on strategy to evaluate students’ opinions on the placement of end-of-life care education within the curriculum and their experience of having received ELNEC training previously throughout their program.	Conceptual framework not evident. Year of students’ program: Bachelor of Nursing Science students. Facilitation not evident. Delivery by film observation simulation experiences, companion programs, specific assigned readings, role playing, and journaling/writing for reflection.	Ethics approval of the institutional review board. Mixed method teaching evaluation study. A 12-question survey used mixed methods to evaluate 37 students’ opinions.	Preparedness and implication on practice not evident. However, study revealed that students’ attitude toward death and dying can be improved through education.	EOL education at the BSN level should be informed by students’ previous knowledge, preparation, and clinical practice exposure. EOL care contents required focused curriculum planning. A constructivist educational model and reflective teaching methods were recommended for earlier-stage students. Incorporating simulation into the nursing curriculum is better than classroom instruction only.
[47] Lippe et al., (2017) Students preparation to care for dying patients. United States of America.	To examine the differences in student outcomes regarding EOL care within and between subgroups of nursing students in a single program.	Content, Input, Process, and Product (CIPP) Model. Year of students’ program, facilitation and delivery not evident.	Study approved by the university’s institutional review board. An exploratory, descriptive, observational study design involving cross-sectional surveys. Sample: five sub-groups of nursing students in a BSN program. Intervention: Content, Input, Process and Product (CIPP).	Students experience positive changes in knowledge and attitudes regarding EOL care as they progress through the curriculum.	It was recommended that education is important to prepared nursing students to provide safe, competent, evidence-based, compassionate care that facilitates individuals experiencing a peaceful death.
[48] Lippe and Carter (2015) End-of-life care teaching strategies in prelicensure nursing education. United States of America.	Focus on characteristics of end-of-life teaching strategies and their impact on student educational outcomes	Conceptual framework N/A. Year of students’ program: Prelicensure nursing students. Facilitation and delivery not evident.	Integrative review. Timeline not evident. 14 papers. Method of analysis not evident.	Nursing students benefit from receiving end-of-life care education in their prelicensure curriculum.	Better quality nursing research on effective teaching strategies in end-of-life care nursing education is needed to ensure that all students receive sufficient education to provide safe and effective care to dying patients.
[49] Mason et al., (2020). A multimodality approach to learning: educating students in palliative care. United States of America.	To examine the effect of educating nursing students utilizing an interactive, multimodality palliative care class that focuses on palliative and end-of-life care.	Kolb’s Experiential Learning Theory Year of students’ program and facilitation not evident. Delivery by lectures, games, simulation, small group discussions, computerized learning modules, readings, and reflection (this includes assignments on students’ observation experience and an analysis of a palliative care case study).	Ethical approval from internal review board. Pre and post-test experimental design. Sample includes undergraduate and graduate level nursing students newly enrolled. An electronic survey via Qualtrics was used. Palliative Care Quiz for Nursing (PCQN) used quasi-experimental design to evaluate knowledge before and after the palliative care class. Intervention: Kolb experiential learning theory.	Preparedness and impact on practice not evident. However, Pre-test results demonstrated a lack of knowledge, attitude, and comfort level palliative and EOL care among the students. While post-test demonstrated improved knowledge of palliative care and symptom management strategies.	Emphasis on symptom management plus additional practicum hours through a combination of clinical and simulated experiences would help improve student comfort and competency in palliative care.
[50] Mazanec et al., (2019) educating associate degree nursing students in primary palliative care using online elearning. United States of America.	Focuses on the development and implementation of an innovative online nursing curriculum that prepares nursing students with the essential primary palliative nursing skills needed by graduation.	Conceptual framework, year of students’ program, facilitation and delivery not evident.	Discussion paper.	Preparedness and implication on practice not evident.	Need for overall curriculum revision to accommodate the six one-hour online modules. Students should be given the opportunity to debrief about the sensitive content by integrating rich discussions into clinical conferences.
[51] O’Connor, B. (2016) CARES: Competencies and recommendations for educating undergraduate nursing students preparing nurses to care for the seriously ill and their families. United States of America.	Aim not evident. A documentary paper that emphasises the essential statement grounded in the mandates for educating nurses in quality, safe, and interprofessional team-based care.	Conceptual framework, year of students’ program, and facilitation and delivery not evident.	Ethics N/A Documentary review. A roundtable of expert nurses and other health care professionals to stimulate scholarly dialog and recommendations on the care of patients at the end-of-life.	Preparedness and impact on practice not evident.	Embedding agreed competencies in the undergraduate curriculum, will empower future nurses to be leaders in advocating for access to quality palliative care and to compassionately promote and provide this essential care.
[52] Pereira et al., (2021). Nursing education on palliative care across Europe: results and recommendations from the EAPC taskforce on preparation for practice in palliative care nursing across the EU based on an online-survey and country reports. Portugal.	To describe current undergraduate and postgraduate nursing education across Europe. To identify the roles that nurses with different palliative care educational levels have in palliative care. To assess the uptake of the EAPC 2004 Guide in the development of palliative care nursing in Europe.	Conceptual framework, year of students’ program, facilitation and delivery not evident.	Ethical approval from EAPC board of directors. Descriptive research involving an online survey. 135 expert nurses from 25 countries.	Preparedness and impact on practice not evident. Study is on the EAPC 2004 document on palliative care nursing education used in many countries to foster and influence the development of palliative care nursing education.	It was recommended to focus mainly on fostering the use of the EAPC 2004 guide on palliative care education and implementing policy measures to ensure recognition and certification the specialty in all European countries. Further research and policy initiatives are needed to better relate required nursing competencies with teaching contents and hours in undergraduate and postgraduate programmes.
[53] Ramjan et al., (2010) Integrating palliative care content into a new undergraduate nursing curriculum: the University of Notre Dame, Australia—Sydney experience. Australia.	To describe how palliative care content has been embedded throughout the three-year University of Notre Dame Australia, Sydney (UNDA) undergraduate nursing degree.	Conceptual framework N/A. Year of students’ program: Three- year undergraduate nursing curriculum. Facilitation by palliative care teaching expertise. Delivered by virtual lectures, tutorial, and face-to-face clinical scenario, clinical practice units, and clinical placements, theoretical and experiential learning.	Discussion paper.	Preparedness not evident. There was limited research about the timing and teaching of palliative care content in undergraduate courses.	Palliative care undergraduate nursing learning needs are best addressed through a multifaceted approach, conducted over several weeks, with a mix of didactical methods and exploration of multiple themes which are integrated with practical experiences and then reflection.
[54] Robinson, (2004) End-of-life education in undergraduate nursing curricula. United States of America.	To discuss the importance of including EOL content in nursing curricula.	Conceptual framework, years of students’ program, facilitation and delivery not evident.	Quantitative study. Ethics and sample N/A. Data collection: A survey conducted.	Many nursing curricula are already crowded and faculty feel ill prepared to teach EOL education.	EOL competencies be incorporated into existing nursing courses
[55] Thrane, (2020) Online palliative and end-of-life care education for undergraduate nurses United States of America.	To describes the development and delivery of an undergraduate level online nursing elective course in palliative and end-of-life care offered through a large public university.	Conceptual framework not evident. Year of students’ program: baccalaureate students. Facilitation by instructors. Delivered by an innovative learning strategy through an asynchronous online format that uses discussion, reflective essay, assignments, difficult conversation, serious games, readings, short taped lectures, and multi-media content.	Discussion paper.	Both the instructor and the students felt that the course was a success.	To cover all concepts in modules throughout the curriculum, a standalone course like the one covering specific palliative and end-of-life content should be required in all baccalaureate nursing programs
[56] William et al., (2021) Global Initiation of palliative nursing education to improve health crisis preparedness. United States of America.	To first provide an overview of palliative care and its importance in serious illness care. To describe how the consequences of COVID-19 require a palliative care perspective using the U.S.-based National Consensus Project (NCP) Clinical Practice Guidelines for Quality Palliative Care readily applicable to the global arena.	Conceptual framework, year of students’ program and facilitation not evident. Delivery by online curriculum or as a hard copy.	Discussion paper. Timeline, number of papers reviewed and method of analysis not evident.	Preparedness and impact on practice not evident. However, Review of the National Consensus Project Clinical Practice Guidelines for Quality Palliative Care and use of educational exemplars highlight opportunities for improving palliative nursing education in academic and clinical settings.	Provision of universal palliative care is ethically mandate for all health systems. Nurses worldwide must be equipped through education to ensure the delivery of palliative care at primary and specialty levels. Global curricular integration of palliative care is needed urgently to properly prepare nursing workforce and provide holistic, person-centred care for patients and their families throughout the continuum of care.
[57] Wilson et al., (2011) An Examination of palliative or end-of-life care education in introductory nursing programs across Canada. Canada.	To assess, describe, and compare PEOLC education across Canadian undergraduate nursing programs.	Conceptual framework, year of study and facilitation not evident. Delivery by lectures followed by small group discussion, and case study as the teaching/learning strategies.	Ethics approval obtained from the Research Ethics Board. A descriptive/ comparative data analysis using SPSS. 35 Nursing schools/ Faculties across Canada. Survey via email.	Preparedness and implication on practice not evident. Lack of time in the curriculum was the most frequently cited followed by a lack of clinical placement or practice options and a lack of knowledgeable teachers.	Educators are encouraged to have a more in-depth understanding of the needs of dying persons and their families. Death education should be included in introductory nursing programs and it need to be more emphasized in the future.

**Abbreviations**: AANC–America Association of Nursing Council, ANOVA- Analysis of Variance, BSN- Bachelor of Science in Nursing, CIPP- Content, Input, Process and Product, EAPC- European Association of Palliative Care, ELNEC- End-of-Life Nursing Education Consortium, EOL- End-of-Life. FATCOD- Frommelt Attitude Towards Care of the Dying Scale, IPE- Interprofessional Education, PC- Palliative Care, PEOLC-Palliative and End-of-Life Care, PCQN- Palliative Care Quiz for Nursing, SPSS- Statistical Package for Social Sciences, STATA- Statistical Software UG-Undergraduate, UNDA- University of Notre Dame Australia, UNPCKS- Undergraduate Nursing Palliative Care Knowledge Survey.

### Arranging, summarising and communicating the outcomes

The fifth and final stage of Arksey and O’Malley’s [18] framework involved summarising and communicating the findings. A total of 34 papers was generated representing 10 countries in this scoping review. The papers are summarised and communicated under the study characteristics and the four key objectives identified in step one of the review processes. To support this process the data is mapped and charted within each reported section to present a clear and succinct summary of the data.

## Results

**Fig 1:**
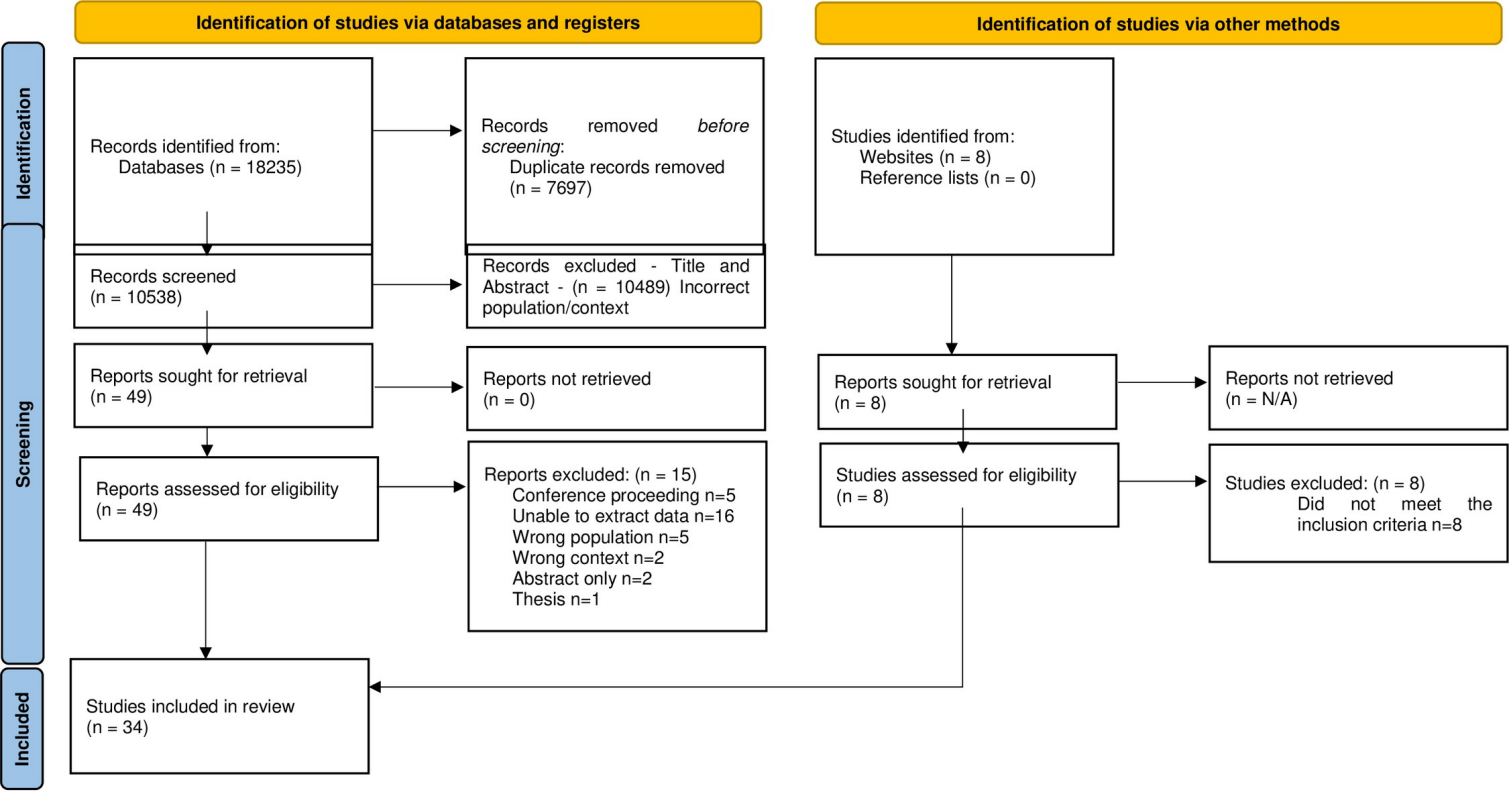
PRISMA flow diagram. From: Page MJ, McKenzie JE, Bossuyt PM, Boutron I, Hoffmann TC, Mulrow CD, et al. The PRISMA 2020 statement: an updated guideline for reporting systematic reviews. BMJ 2021;372:n71. Doi: 10.1136/bmj.n71. For more information, visit: http://www.prisma-statement.org/.

The search of the databases and grey literature generated 18,243 results of which 18,235 originated for the five databases and 8 from grey literature searches. 7,696 duplicates were identified and removed and the remaining 10,547 papers went forward for title and abstract screening. Following title and abstract screening 10,498 papers were excluded leaving the remaining 49 papers going forward to the full-text review stage. The full text review process identified 34 papers that met the inclusion criteria and the reasons for the 15 excluded papers are reported in the PRISMA flow diagram [58] (Fig 1). This review is reported in line with the Preferred Reporting Items for Systematic Reviews and Meta-analysis for Scoping Reviews (PRISMA-ScR) (S1 Checklist) [59].

### Characteristics of the studies

The screening process generated thirty-four papers that met the inclusion criteria from ten countries and more than a half 56% (n = 19) were from the USA. Papers in this review comprised of fourteen secondary data papers, thirteen quantitative papers, four qualitative papers, and three mixed-methods papers (Table 4). The quantitative designs varied, using descriptive, observation, exploration and quasi-experimental designs. Three of the thirteen quantitative papers utilised standardised survey tools: Undergraduate Nursing Palliative Care Knowledge Survey (UNPCKS) [30], Frommelt Attitude Toward Care of the Dying Scale (FATCOD) [38] and Palliative Care Quiz for Nursing (PCQN) [49]. The four qualitative papers utilised qualitative descriptive design [26], ethnography observation [39], qualitative approach and semi-structured interview [45] and consensus-based design [51]. One of the three mixed-method papers utilised a longitudinal quantitative approach and thematic analysis to analyse qualitative data in response to open ended questions [36]. The second utilised a consultation survey via telephone and email, and a consensus-based comment and national approval process for data analysis [40]. The third paper used a 12-question survey that utilised mixed-method approach to evaluate students [46]. In addition, within the discussion/opinion papers one utilised reflective essay and assignment [46, 49, 55]. One utilised 2 to 3 nursing licensure examination interwoven throughout the course and a 10-question quiz with nursing licensure type items [33]. A third utilised an observer checklist in debriefing sessions [14] to evaluate learning outcome.

**Table 4:** Characteristics of the study.

Country	n =	Paper
United States of America	n = 19	[25–30, 33, 34, 44, 47–51, 54].
United Kingdom	n = 4	[14, 36, 38, 52].
Canada	n = 2	[40, 57].
Australia	n = 2	[42, 53].
China	n = 2	[41, 46].
New Zealand	n = 1	[37].
Egypt	n = 1	[32].
India	n = 1	[43].
Brazil	n = 1	[45].
Uganda	n = 1	[31].
**Design**	**n =**	**Paper**
Quantitative	n = 13	[29, 30, 32, 37, 38, 41–43, 47, 49, 52, 54, 57].
Qualitative	n = 4	[26, 39, 45, 51].
Mixed-method	n = 3	[36, 40, 46].
Discussion/opinion	n = 14	[14, 25, 27, 28, 31, 33–35, 44, 48, 50, 53, 55, 56].

The sample size of the quantitative papers in this review consists of the undergraduate student nurses, nursing schools/faculties, nurse educators/experts, academic leads and deans of nursing schools. Surveys included 15 national nursing faculties [29], 35 nursing schools/faculties [56] 88 nurse educators and 135 expert nurses [32], 13 academic leads and 36 deans of nursing schools [37, 42]. However, other papers did not reference the number of respondents [30, 48, 49, 53]. Student sample sizes within the quantitative papers spanned from 83 third-year diploma nursing students [43] to 117 third-year nursing students [38] and 1,200 undergraduate nursing students [28]. Within qualitative papers sample sizes spanning from 13 participants (7 newly trained nurses and 6 nurse tutors) [45] to 17 female junior and senior baccalaureate students [26] and 60 third-year nursing students in eight group sessions [39]. The mixed-methods papers had sample sizes of 336 year-three student nurses at one university [36], 37 bachelor of nursing students [46] and 8 faculties of Canadian schools of nursing [40]. The samples represented in this review were characterised by a wide range of ages, nursing programs and ethnicities with degree program students as the majority in representation.

### What educational models are utilised within palliative care undergraduate student nurse education?

Four educational models were identified in this review and the conceptual or theoretical framework underpinning the palliative care education approach was evident in 5 papers and students’ year of study evident in 12 papers (Table 5). Within the papers reviewed, the facilitators delivering the palliative care education were evident in 11 papers and educational delivery strategies in 22 papers (Table 6). While a broad range of educational facilitators are identified it is recommended that palliative care education should be facilitated by experts [25, 32, 39, 57] and that experts are needed for high-fidelity simulation to enhance knowledge in palliative care and mentor students [32, 44].

**Table 5:** Educational characteristics.

Education model	n =	Paper
Palliative care (PC) education.	N = 16	[14, 30, 31, 34, 35, 40, 41, 43–45, 49–52, 54, 56].
End-of-Life Care (EOLC) education.	N = 11	[25, 27–29, 33, 36, 39, 46, 48, 54, 55].
Death and dying education.	N = 4	[26, 37, 42, 47].
Palliative and End-of-Life care (PEOLC) education.	N = 3	[32, 37, 57].
**Conceptual Framework**	**n =**	**Paper**
Share Theory of Palliative Care	n = 1	[30].
Kolb Experiential Learning Theory	n = 1	[49].
Scaffolded approach	n = 1	[36].
Morin’s Theory of Complexity Hologrammatic Principle of Complex Thinking	n = 1	[45].
Content, Input, Process, and Product (CIPP) Model	n = 1	[46].
**Year of student engagement**	**n =**	**Paper**
Third-year nursing students	n = 3	[36, 38, 39].
Junior and senior baccalaureate students. Baccalaureate students. Third year baccalaureate students	n = 3	[26, 30, 55].
Pre-licensure nursing students	n = 2	[33, 48].
Bachelor of nursing students undergraduate nurses	n = 2	[25, 46].
Third-year diploma nursing students	n = 1	[43].

**Table 6:** Facilitation and delivery characteristics.

**Facilitation**	**n =**	**Paper**
Nurse educators/tutors	n = 3	[30, 32, 39].
Academic nurses with contributions from other professionals	n = 1	[37].
Clinical instructors	n = 1	[55].
Palliative care nurse leaders	n = 1	[33].
Teacher led	n = 1	[35].
Nurse educators and expert guess lecturers	n = 1	[28].
Palliative care teaching expertise	n = 1	[53].
Registered nurses from the palliative care clinical setting	n = 1	[42].
Guests’ lecturers, religious leader, ethicist, social worker, counsellor, psychotherapist, morticians, nurses, physicians, lawyer, caregivers, and classmate from a different culture	n = 1	[26].
**Delivery strategy**	**n =**	**Source**
Both didactic and experiential	n = 3	[14, 26, 42].
Simulation	n = 2	[38, 39].
Online platform interactive technology	n = 1	[29].
Lecture pedagogy teaching strategy, clinical field practice, and group discussion.	N = 1	[32].
Lectures, tutorials clinical placements, case-based teaching, simulation and patient presentation/interview.	N = 1	[37].
Online curriculum or as a hard copy	n = 1	[56].
Lectures, games, simulation, small group discussions, computerized learning modules, readings, and reflection	n = 1	[49].
An asynchronous online format that uses discussion, reflective essay, assignments, difficult conversation, serious games, readings, short taped lectures, and multi-media content	n = 1	[55].
Pedagogical approaches to encourage problem-based learning, facilitating role play and exploration of audio-visual resources (simulation)	n = 1	[36].
Case studies with critical-thinking reflection, brief videos demonstrating key palliative nursing skills.	N = 1	[33].
Film observation simulation experiences, companion programs, specific assigned readings, role playing, and journaling/writing for reflection.	N = 1	[46].
Face-to-face instruction, clinical or practicum experience online teaching, and simulation	n = 1	[27].
Lecture, simulation and experiential	n = 1	[25].
Lecture, seminar/ small group format, role-play, videos case studies, and visits to local hospices and/or terminal patient visits and online format (simulation).	N = 1	[26].
Experiential activities, including touring the local hospice care centre and retirement community	n = 1	[35].
Simulation (high-fidelity simulators/role play/actors).	N = 1	[44].
Lectures followed by small group discussion, and case study	n = 1	[57].
Lectures, tutorial, and face-to-face clinical scenario, clinical practice units, and clinical placements, theoretical and experiential learning.	N = 1	[53].
Reflection on issues	n = 1	[45].

### What methods have been used to assess effectiveness of palliative care undergraduate student nurse education?

Only 4 papers in this review highlights the evaluation of learning outcomes. The first paper utilised 2 to 3 nursing licensure examination interwoven throughout the course and a 10-question quiz with nursing licensure type items [33]. The second paper utilised an observer checklist within debriefing sessions [14]. The third paper utilised an assignment on student observation experience and case study analysis of a palliative care case study with pre and post-test [49]. The fourth paper utilised a reflective essay and assignment [55]. Within this review, palliative care education was deemed effective if it resulted in positive learning outcomes for students and 15 papers highlight effectiveness which was identified through knowledge gained, self-confidence and adequate preparedness (Table 7).

**Table 7:** Effectiveness.

**Effectiveness through knowledge gained**	**n =**	**Paper**
Highlight opportunities for improving knowledge through palliative nursing education in academic and clinical settings	n = 1	[56].
Post-test demonstrated improved knowledge of palliative care and symptom management strategies.	N = 1	[49].
Both the instructor and the students felt that the course improved knowledge and was a success	n = 1	[55].
Multiple methods of education delivery have shown to increase nursing students’ knowledge and positive attitude about EOL care	n = 1	[25].
The use of live actors and role play were effective in increasing student knowledge and self-efficacy	n = 1	[44].
Students experience positive changes in knowledge and attitudes regarding EOL care	n = 1	[47].
Nursing students benefit from receiving end-of-life care education in their prelicensure curriculum	n = 1	[48].
Majority of the students expressed the usefulness, realistic and a good learning opportunity to handle challenging communication from patients and family members.	N = 1	[39].
Students gained more knowledge in helping patients, families, and themselves in end-of-life care related issues	n = 1	[26].
**Effectiveness through self-confidence and adequate preparedness**	**n =**	**Paper**
Early integration impacted student perceived preparedness and attitudes positively	n = 1	[29].
EAPC 2004 document on palliative care nursing education used in many countries to foster and influence the development and preparedness of students for palliative care nursing education	n = 1	[52].
Students identified growth in their practice, confidence and preparedness to deliver person-centred care in end-of-life-care	n = 1	[36].
Students have primary palliative care contents in their curricula and they felt more prepared to care for patients and families, especially at the EOL	n = 1	[33].
Simulation was said to adequately prepared student nurses for EOL care. Structured palliative care course influenced students’ ability to be clinically competent	n = 1	[38].
Educational process in a PC education has contributed to preparing students for the care of terminally ill patients	n = 1	[45].

### What facilitators/barriers have been reported relating to the success/failures of the models of palliative care undergraduate student nurse education?

Within this review nine of thirty-four papers reported a wide range of facilitators that contributed to the success of the models of palliative care undergraduate student nurse education. The educational process was highlighted as a means of preparing students for care of the terminally ill [45]. While early integration was highlighted as enhancing and impacting undergraduate student nurses’ comfort and perceived preparedness [29]. Multiple learning methods have been shown to increase knowledge and positive attitude of undergraduate student nurses towards the provision of palliative care [54] and clinical exposure to meaningful learning opportunities with patients experiencing serious life-threatening illnesses facilitates learning and enables change of attitudes [32]. A structured palliative care course [38] and the use of palliative care documents and guidelines to facilitate the success of palliative care education within the undergraduate student nurses’ program were seen to influence students’ ability to be clinically competent [52, 56]. In the last decade, the use of simulation and analysis of scenarios has assisted to prepare students [38] and a key element within the simulation was debriefing which allowed for analysis of the dynamics occurring during the scenario [25].

Within this review, several factors acted as barriers to palliative care education were evident specifically the issue of a crowded curricula and the lack of time in the curriculum [40, 53, 57]. This lack of time and crowded curriculum is compounded by the lack of palliative care expertise [40] and the difficulty in providing clinical placement [57]. This results in educators and students feeling ill prepared to deliver palliative care education or feel adequately prepared for future practice [40, 54]. Furthermore, there is limited research about the timing and teaching of palliative care content in undergraduate course [44] and students can find simulations using manikins difficult [47]. These factors limit undergraduate student nurses in developing the cognitive skills required for effective palliative care clinical decision making [44].

### What gaps in the literature exist on preparation within palliative care undergraduate student nurse education?

In this review there is evidence of disparity and lack of research in low-middle income countries. Also, there is evidence specific to undergraduate student nurse palliative care education preparedness, barriers and implications for practice in this review. From the educational aspect, there is a lack of integration of palliative care philosophy and conceptual framework [44], crowded curricula [53], lack of nurse educators and expertise to teach palliative care within nursing faculties [40, 53, 57] and timing and teaching of palliative care [44] were evident from the empirical studies in this review. From a clinical practice perspective there are difficulties in providing clinical placement [57] and responding to manikins presents difficulties [47] and these barriers warrant further investigation. Hence, there is a need for further and continual research and publications in many areas of palliative care education.

## Discussion

This scoping review draws together the research literature on undergraduate student nurse education and preparation for palliative care. The focus of papers identified in this review were on palliative care education (sixteen papers), end-of-life care education (eleven papers), death and dying education (four papers) and palliative and end-of-life care education (three papers). These are specific terms used for people living with life-limiting conditions that require palliative care depending on the stage of their illness or condition. The papers reviewed highlight that palliative care undergraduate nurse education is evident in both high-income countries (twenty-nine papers 85%) and in low- and middle-income countries (five papers 15%). Notwithstanding, the impact of globalisation and the effect the internet and technology has on influencing and sharing information world-wide, it is evident from this review that there is a lack of published research from low- and middle-income countries or collaborative palliative care education or research between high and low- and middle-income countries. This is important as the highest proportion of adults in need of palliative care are from low- and middle-income countries and where palliative care still developing and is primarily limited to urban areas [60].

In discussing the review question (a) What educational models are utilised within palliative care undergraduate student nurse education? This review highlights that theoretical and experiential learning go hand-in-hand in palliative care education and training [38]. This review identified five conceptual frameworks for palliative care education see Table 5. Utilising frameworks in education provides a fundamental structure to explain human behaviour towards learning [61]. Educational models are the philosophical foundation of any overall approaches and beliefs about learning, instruction and content through providing meaning and direction [62]. However, there was no evidence of integration of palliative care philosophy with the conceptual frameworks identified in this review. The use of a conceptual framework to deliver palliative care is a valuable tool for nurse educators to structure education and serves as a standard for practice because many educators/teachers continue to find values and benefits in using learning styles concept that are applicable in different situations. There is likely to be a conceptual framework which is appropriate in one situation and not in another. Therefore, there is a need to be clear which palliative care educational models and the methods are beneficial and appropriate to use. Following which it is important to investigate the conceptual frameworks appropriates and fit with palliative care philosophy to facilitate, deliver and evaluate palliative care education for undergraduate student nurses.

Discussing the review question (b) What methods have been used to assess effectiveness of palliative care undergraduate nurse education? This review highlights that palliative care educational programs facilitated by an expert multi-disciplinary team through a series of course contents that are both theoretical (classroom) and practical (skills practice in a simulated setting with anatomic models) is most effective and promote better preparation of undergraduate student nurses. For palliative care education to be effective it must result in positive learning outcomes for students [63]. Within this review effectiveness was identified through knowledge gained, self-confidence and adequate preparedness and is in line with other research [41, 64]. Key within palliative care education is assessment of learning as it determines whether the learning goals are met. Well-designed assessment methods provide valuable information about student learning and identifies what was learned, how well and where they struggled [52]. The assessment methods identified in this review seem limited when compared to palliative care philosophy and educational contents. Hence, there is a need for further investigation of assessment methods that identify learning, preparedness, competence and outcomes in practice. In some incidents funding is required to determine the effectiveness of palliative care learning outcomes especially in low- and middle-income countries.

Discussing review question © What facilitators/barriers have been reported relating to the success/failure of the models of palliative care undergraduate student nurse education? It is evident that palliative care education is facilitated by a multidisciplinary team that includes nurse tutors, palliative experts, expert guest lecturers and other professional experts. Both theoretical and experiential components develop students’ knowledge, skills, and attitudes. Additionally, palliative care education is being delivered using single or multiple strategies in the classroom, online and in a simulated environment/skills development laboratory, where students continue to practice key skills and demonstrate attitudes in a supervised clinical setting.

The use of blended learning that works together to develop students’ knowledge, skills, and attitudes is evident in this review. Educational processes which organise and systematise patient care [45, 52] and early integration [29, 46] enhance and impact undergraduate student nurses’ comfort and perceived preparedness. Multiple learning methods have shown to increase knowledge and positive attitude of undergraduate student nurses towards the provision of palliative care [54, 65] and clinical exposure to meaningful learning opportunities facilitates learning [32, 66]. Structured palliative care course influence students’ ability to be clinically competent [38] and the use of palliative care documents and guidelines facilitates the success of palliative care education within the undergraduate student nurses’ program [6, 7]. In addition, within the last decade the use of simulation and analysis of scenarios has assisted to prepare students [38, 63] and a key element within simulation is debriefing which allows for analysis of the dynamics occurring during the scenario [25, 67].

What is evident from this review and the wider literature is that palliative care education requires careful planning, selection of appropriate teaching methods and learning materials, developing a course schedule and planning for student assessment [27, 68]. In addition, palliative care educational programs typically require a period of study, allowing time and opportunities for students to develop essential competencies that encompass essential knowledge, skills, values, and positive attitudes [69]. Therefore, palliative care undergraduate student nurse education should start early in the program and it is crucial for nurse educators to carefully define the core knowledge that students must achieve at each level of their academic program [7, 29, 46].

However, to achieve the delivery of palliative care education within undergraduate nursing programs a balance has to be achieved to offset the barriers of a crowded curricula and the lack of time in the curriculum [29, 40, 53]. This lack of time and crowded curriculum is compounded by the lack of palliative care expertise [40]. and the difficulty in providing clinical placement [57]. These result in educators feeling ill prepared to deliver palliative care education and students feeling inadequately prepared for future practice [70]. Furthermore, this review highlights that there is limited research and agreement about the timing and teaching of palliative care content in undergraduate course [44, 71] and simulation presents difficulties for students [47, 72].

While a broad range of facilitators are identified within this review [25, 29, 39, 57] it is recommend that palliative care education be facilitated by experts to enhance knowledge in palliative care and mentor students particularly for high fidelity simulation [73]. The absence of expert facilitation may limit undergraduate student nurses development of the cognitive skills required for effective palliative care clinical decision making [44, 74]. Therefore, there is a need for guidance and support for both educators and students in simulation laboratory [75]. To support nurse educators responsible for designing undergraduate student nurse course contents expertise and training should be both theoretically and practically to adequately prepare students for practice.

Discussing review question (d) What gaps in the literature exist on preparation within palliative care undergraduate student nurse education? The evidence from this review highlights the disparity and lack of research in low- and -middle income countries. Research efforts build the science of nursing education through the discovery and translation of innovative evidence-based strategies. Therefore, there is a need for nurse educators, researchers and experts in the field of palliative care in low- and -middle income countries to be involved in rigorous research in the teaching-learning process and outcomes at all levels of nursing education. From the educational perspective, this review highlighted the integration of palliative care philosophy and conceptual framework, crowded curricula, the lack of nurse educators with expertise and delivery timing of palliative care education as areas for further consideration and investigation.

Given that palliative care education improves students’ knowledge and increases their preparedness, further research is warranted into the correlation of palliative care philosophy and the conceptual framework to underpin undergraduate student nurse education. From a practice perspective the difficulty in providing clinical placement needs consideration and investigation. To address placement issues there is a need to consider and improve on the limited clinical placement on specialist unit, large number of students scheduled for placement at the same time and short length of placement that may negatively impact the opportunities for clinical experiences in real patients care situations. In addition, family/client involvement in palliative care education and placement provision needs to be addressed in the context of future service provision and the rights of people with life-limiting illness concerning choices that affect their lives. This needs to be considered in terms of how undergraduate student nurses will meet the professional training and educational standards requirements upon graduation. The consideration of a hub and spoke model could be one way to of bridging this issue and utilising family placement as a learning environment for students for preparing them for their future role as a registered nurse [76, 77].

### Limitations

While this review used precise, transparent methods based on study and reporting guidelines by Arksey and O’Malley [18] no quality appraisal was conducted as the focus of this review was to update and map the evidence. Thus, this paper only offers a descriptive account of available information and there was no patient and public involvement and there are opportunities for engagement, potentially following published guidance on stakeholder involvement in systematic reviews [78]. In addition, papers in this review were limited to only five databases which may have affected the inclusion of low- and -middle income countries and the inclusion of secondary data can be seen as both a strength and limitation.

## Conclusions

This scoping review illustrates the evidence on palliative care education in preparing undergraduate student nurses for practice. The evidence identifies a wide, varied and expanding range of educational models, facilitation and delivery strategies currently in use. It is evident from this review that undergraduate student nurses require palliative care education in order to produce nurses with the graduate capabilities to deliver high-quality palliative care and to better meet the needs of an ageing population and rising consumer expectations. However, it is suggested that the integration of palliative care education contents requires focused curriculum planning to ensure clarity and cohesion in the content delivery method that is interactive and flexible involving application of different teaching strategies to minimise gaps and overlaps. While effectiveness of palliative education is important as it increases students’ knowledge, improves their attitudes and prepares them to provide palliative care, future research needs to measure application to practice. Furthermore, future research needs to detail the content delivery method, assessment and evaluation method clearly.

## Supporting information

S1 Checklist Preferred Reporting Items for Systematic reviews and Meta-Analyses extension for Scoping Reviews (PRISMA-ScR) checklist. (DOCX)
